# Glucocorticoid Effects on Tissue Residing Immune Cells in Giant Cell Arteritis: Importance of GM-CSF

**DOI:** 10.3389/fmed.2021.709404

**Published:** 2021-09-07

**Authors:** Annette D. Wagner, Ulrike Wittkop, Jessica Thalmann, Tina Willmen, Vega Gödecke, Justyna Hodam, Simon Ronicke, Martin Zenke

**Affiliations:** ^1^Department of Nephrology and Hypertension, Hannover Medical School, Hannover, Germany; ^2^Deutsches Rotes Kreuz (DRK) Clinic for Child and Adolescent Psychiatry, Bad Neuenahr, Germany; ^3^Department of Cell Biology, Institute for Biomedical Engineering, Rheinisch-Westfälische Technische Hochschule Aachen (RWTH) Aachen University Medical School, Aachen, Germany; ^4^Helmholtz Institute for Biomedical Engineering, Rheinisch-Westfälische Technische Hochschule Aachen (RWTH) Aachen University, Aachen, Germany

**Keywords:** glucocorticoids, dendritic cells, giant cell arteritis, GM-CSF, apoptosis

## Abstract

Giant cell arteritis (GCA) is a systemic granulomatous vasculitis clinically characterized by a prompt response to glucocorticoid therapy. Dendritic cells (DCs) play a central role in the pathogenesis of the disease and are increased in temporal arteries from GCA patients. The aim of this study was to determine the effects of glucocorticoid therapy on granulomatous infiltrates and on peripheral DCs of GCA patients. Immunohistochemical staining of temporal artery specimens from 41 GCA patients revealed a rapid reduction of the number of DCs after initiation of glucocorticoid treatment. TUNEL staining was performed to quantify apoptotic S100+ DC, CD3+ T cells, and CD68+ macrophages in the granulomatous infiltrates. An increase of apoptotic cells up to 9 ± 2% after 4–5 days of glucocorticoid therapy and up to 27 ± 5% (*p* < 0.001, compared to earlier timepoints) after 6–10 days was detected. A decrease of CCL19 and CCL21 expression was observed after starting glucocorticoid therapy. Granulocyte-macrophage colony-stimulating factor (GM-CSF) expression also significantly decreased under glucocorticoid therapy. No GM-CSF expression was detected in the control specimens. Glucocorticoid therapy leads to a rapid, time-dependent reduction of DCs in temporal arteries from GCA patients and reduction of mediators for cell migration. Our data suggest GM-CSF as a novel therapeutic target of GCA.

## Introduction

GCA is a vasculitis predominantly affecting medium- and large-sized arteries. DCs play a significant role in the pathogenesis of large vessel vasculitides. Studies of large-vessel vasculitis have shown that activation of tissue residing DCs causes T cell and macrophage activation that subsequently leads to granuloma formation in the vessel wall (1). DCs are found at the media-adventitia junction where they act as pathogen sensors (2). Previous data showed the immediate neighborhood of DCs and activated CD4+ T cells in inflammatory lesions of temporal artery specimens from GCA patients indicating that there was a high probability of DCs being the key antigen presenting cells in GCA (3). It was shown that activated DCs stimulate autologous CD4 T cells, which produce the proinflammatory cytokine interferon-gamma (IFN-γ) and infiltrate deeply into the vascular smooth muscle cell layer, causing matrix damage (4). Granulomatous lesions in inflamed temporal arteries harbor an array of cytokines and inflammatory mediators (1). There are two distinct immunological pathways: the interleukin (IL)-12–type 1 helper T-cell (Th1)–IFN-γ axis and the IL-6–type 17 helper T-cell (Th17)–IL-17 or IL-21 axis (5). There is evidence that Th1-related immune responses are not effectively targeted by glucocorticoid treatment (6). In fact, Th17 related cytokines have been shown to hamper both anti-inflammatory and immunosuppressant actions of glucocorticoids in peripheral lymphocytes via increased glucocorticoid-receptor beta expression (7).

Most DCs in peripheral tissues have an immature phenotype. After detection of microbial products in the presence of proinflammatory cytokines, immature DCs start to develop into mature DCs. Mature DCs develop an exceptional capacity for T cell stimulation. Immature DCs in healthy arteries fail to stimulate T cells, but DCs in polymyalgia rheumatica arteries have shown to activate T cells that originated from the GCA lesions. Therefore, it was proposed that *in situ* maturation and activation of adventitial DCs initiate and maintain T cell responses in GCA (8).

There are several factors influencing this process. Chemokines are known to be involved in the pathogenesis of GCA and are crucial for the activation of DCs. It was shown that in fully developed GCA, CCL19 expression in the vessel wall was increased 8-fold above controls and CCL21 expression was 24-fold higher than in normal arteries. In immunohistochemical studies, the major source of CCL19 and CCL21 protein was found to be CD83+ DCs (8). Also, the granulocyte macrophage colony stimulating factor (GM-CSF) is an important hematopoietic growth factor and immune modulator (9). Compared to patients with GCA in remission, patients with active GCA have increased mean levels of GM-CSF in culture supernatants of stimulated PBMCs (10).

GCA is clinically characterized by a prompt response to glucocorticoid therapy. Glucocorticoids are potent anti-inflammatory and immunosuppressive agents that act on different cells of the immune system, including T cells, monocytes, macrophages, osteoclasts and DCs (11–14). It has been shown that in sepsis glucocorticoids act on DCs by suppressing IL-12 production and therefore ameliorate inflammatory overresponse (15).

In monocytes and macrophages, glucocorticoids have the ability to down-regulate the production of a large number of cytokines, including IL-1, IL-6, IL-8, IL-12, tumor necrosis factor alpha (TNF-α), and GM-CSF (16–18). Glucocorticoids have also been reported to induce apoptosis in thymocytes and T lymphocytes (19, 20). Another study showed that glucocorticoids are potent inhibitors of bioactive IL12-p70 heterodimer production by human DCs (21). Earlier investigations indicated that glucocorticoids decrease the migratory capacity of DCs *in vitro* and reduce emigration of leukocytes from vessels (22). Further studies showed that the numbers of DCs in rat airway mucosa decrease rapidly after glucocorticoid treatment (23). It was shown that glucocorticoid treatment causes down-regulation of CD86, CD40, CD54, and MHC class II molecules on DCs (24).

To further investigate the role of DCs in the pathogenesis of GCA it was of major interest to identify the effect of glucocorticoid treatment on DCs in the granulomatous infiltrates in temporal arteries and in peripheral DCs of GCA patients.

## Materials and Methods

### Patients

Temporal artery biopsy specimens for the respective immunohistochemistry, immunofluorescence techniques and in part for *in-situ* hybridization were procured from 41 GCA patients. The tissue samples used in this study were obtained prior to 2007. The tissue samples were taken from patients with a suspected GCA to establish the diagnosis. CD1c+ DC were isolated from 100 ml heparinized peripheral blood from 5 GCA patients. The peripheral blood samples were obtained in the period from 2002 to 2005. Consent by the institutional review board of the Hannover Medical School was given (No. 2752. 22.08.2001).

All the patients fulfilled the American College of Rheumatology (ACR) 1990 criteria for the classification of GCA (25). With the exception of 15 patients, bilateral temporal artery specimens were available from all. From each GCA patient 15 paraffin-embedded tissue sections were obtained: six consecutive samples were selected for TUNEL-Assay and the remaining nine consecutive samples were used for immunohistochemical studies. Temporal artery biopsies of the GCA patients showed typical relevant histopathology.

Patients included in this study were 50 years and older. These patients were treated with glucocorticoids for different time intervals before a biopsy was taken. Eight patients were untreated, 10 patients were on glucocorticoids for 1 day, 14 patients for 2 days, five patients for 3 days, three patients for 4 days, five patients for 5 days, two patients for 7 days, one patient for 10 days, and three patients were on long-term glucocorticoid therapy (longer than 14 days). These groups were selected to analyse the course of treatment since repeated biopsies in the same patient are unreasonable. The GCA patients received prednisolone orally in the following dosage once a day: 80 mg were administered in three patients, 75 mg in one patient, 70 mg in 25 patients, 60 mg in one patient and 30 mg in one patient. Only two patients received 100 mg, one patient 120 mg, five patients 300 mg, and one patient 500 mg soludecortin intravenously. Three patients with GCA were on long-term glucocorticoid therapy with a dosage of 5 mg prednisolone once a day. Samples from 11 patients without GCA served as controls. The control patients fulfilled two of the ACR criteria for GCA and were biopsied because of persisting bilateral headaches. Diagnoses of these 11 patients were: anterior ischemic optic neuropathy (*n* = 2) myocardial insufficiency (*n* = 1), polymyalgia rheumatica (*n* = 3), transitory ischemic attack (*n* = 1), stroke (*n* = 2), infectious diseases (*n* = 2). In 5/11 control patients, biopsy specimens were available from both left and right temporal arteries. Tonsillar tissue served as positive and negative control. GM-CSF detection was carried out in temporal artery biopsy specimens from 22 GCA patients that met 4–5 ACR criteria. Three of them were not treated with glucocorticoids, 10 were treated for 1 day, and nine were treated for 2 days at the time of biopsy. Eight temporal artery biopsy samples from 5 patients without GCA (with 2 ACR criteria) served as control. Demographic data of the patients are demonstrated in Supplementary Table 1. Temporal artery specimens were investigated by different immunohistochemistry and immunofluorescence techniques as well as by *in-situ* hybridization. RNA was isolated from CD1c+ dendritic cells and was used for Real time PCR.

Normal tonsil tissue served as positive and negative control. It was processed and stained at the same time as the arteria samples. Unspecific reaction of the tissue with the secondary antibody was excluded by omitting the primary antibody in the negative control.

### Antibodies and Staining Kits

Polyclonal Rabbit anti-cow S100 (strongly cross-reacts with human S100; DAKO Glostrup, Denmark); goat anti-mouse CCL19/MIP-3β (reacting crosswise with the human CCL19), goat anti-human CCL21/6Ckine, monoclonal mouse anti-human CD163, monoclonal anti-human GM-CSF (all from R&D Systems, Minneapolis, USA); monoclonal mouse anti-human HLA-DR (BD Biosciences, San Jose, USA); Cy2-conjugated goat anti-mouse IgG, Cy2-conjugated goat anti-rabbit IgG (both from Jackson West Grove, USA); Dako EnVision Doublestain System, AEC+ high sensitivity substrate-chromogen system, liquid DAB substrate-chromogen system (all from DAKO Carpinteria, USA); ApopTag Plus Peroxidase *in situ* Apoptosis Detection Kit, ApopTag Red Kit (both from Qbiogene, Illkirch, France).

### Immunolabelling of Tissue Sections

Paraffin-embedded temporal artery specimens were deparaffinized, then microwave-heated in citrate buffer.

S100 was used several times to stain dendritic cells (26, 27). For S100 staining, endogenous peroxidase activity was blocked with hydrogen peroxidase. Tissue sections were then blocked with horse serum and stained with the rabbit anti-S-100 antibody (1:1,000) in combination with Dako EnVision Doublestain Kit and developed with AEC. For each patient and control patient, S100+ cells were counted in three different temporal artery sections at 40 × magnification. Staining with anti-HLA DR, anti-CD163, anti-CCL19, anti-CCL21, and anti-GM-CSF antibodies was carried out analogously.

The number of cells per artery was counted 2-fold under the incident light microscope CK40 (Olympus Hamburg, Germany) using 40 × magnification. Selected specimens were photographed by using the Soft Imaging Software (Olympus Hamburg, Germany).

The statistical evaluation of the results was realized using the program SPSS (Statistical Package for the Social Sciences). In the imaging of DC in relation to the duration of the glucocorticoid therapy all values for the number of DC per biopsy for the same duration of therapy were combined in one group, the mean value with standard deviation was determined and the groups were compared with each other.

The ApopTag Plus Peroxidase Kit was performed according to the manufacturer's instructions on 31 paraffin-embedded consecutive temporal artery specimens to quantify apoptotic cells. The number of apoptotic cells in relation to the total number of cells in the granulomatous lesions of the temporal artery cross section was counted in four different random fields at 100 × magnification.

To visualize the DNA fragmentation in the nuclei, ApopTag Red Kit was applied according to the manufacturer's instructions on 12 paraffin-embedded tissue samples. To distinguish between different apoptotic cell types, sections were additionally stained either with anti-S100 Ab, anti-CD68 mAb, and anti-CD3 mAb in combination with the Cy2-conjugated secondary antibody. All 12 double-fluorescence-stained sections were evaluated by confocal microscopy. The selected tissue sections were visually cut by laser scanning along the z-axis cells in 0.5 μm slices.

For the detection of the intracellular localization of the DNA fragments the Laser Scanning-Confocal microscope MRC-600, Bio-Rad Microscience Ltd, Herts, UK was used. The system works with an argon laser and is connected to the Zeiss Axiovert 35 inverted microscope. The device has two separate detectors (photomultiplier) that can simultaneously convert the light energy of two different fluorochromes into electrical signals and display them on the computer. The special pinhole optic of a confocal microscope allows a point-like focusing in an imaging plane and makes a resolution along the optic (z) axis possible. Thus, cells can be optically sliced and intracellular structures can become visible.

The selected preparations were imaged along the z-axis at a distance of 0,5 um, resulting in 12–15 axial imaging planes per cells. The layer images were made using Comos software and recorded in two-channel imaging mode for both fluorochromes.

In the imaging of the apoptosis in relation to the duration of the glucocorticoid therapy all values for the percentage proportion of apoptotic cells for the same duration of therapy were combined in a group, the mean value with standard deviation was determined and the groups were also compared with each other. As a statistical test for assessing the differences between these defined groups the following tests were used: Bonferroni test for the number of DC and Tamhane test for the percentage of apoptotic cells. Differences were considered significant if the test yielded values of *p* < 0.05, or highly significant if it yielded values of *p* < 0.01.

### *In situ* Hybridization

GM-CSF expression was determined by *in situ* hybridization using DakoCytomation *In Situ* Hybridization Detection System For Biotinylated Probes (DAKO Glostrup, Denmark) according to the manufacturer's protocol. GM-CSF+ cells were counted in two different temporal artery sections at 40 × magnification.

### Isolation of Peripheral CD 1c+ Dendritic Cells, RNA Isolation, and Real-Time PCR for CSF2RB

100 ml of heparinized blood was used to isolate peripheral blood mononuclear cells (PBMC) using Ficoll-Hypaque density gradient centrifugation. Positive selection of CD1c+ peripheral DC was performed by using the CD1c (BDCA-1) Dendritic Cell Isolation Kit (Miltenyi, Bergisch-Gladbach, Germany) according to the manufacturer's protocol. To obtain total RNA, the NucleoSpin RNA II Kit (Macherey-Nagel, Düren, Germany) was applied according to manufacturer's instructions. Residues of genomic DNA in RNA samples were digested with RQ1 (Promega, Madison, USA). For Real-time PCR, 0.6 μg total RNA was subjected to reverse transcription using the Reverse Transcription Core Kit with random hexamers (Eurogentec, Seraing, Belgium) according to manufacturer's instructions. Subsequent real-time PCR was performed in an ABI Prism 7000 (Applied Biosystems, Darmstadt, Germany) with gene-specific primers for the colony stimulating factor 2 receptor beta common subunit (CSF2RB) also of the GM-CSF receptor and the housekeeping gene RPS9 in combination with SYBRGreen chemistry (Eurogentec, Seraing, Belgium). Order and sequence information of the oligonucleotides used for real-time PCR is supplied in Supplementary Table 2. Data were analyzed using Q-gene software.

## Results

### Reduction of DC Under Glucocorticoid Therapy

**Figure 1:**
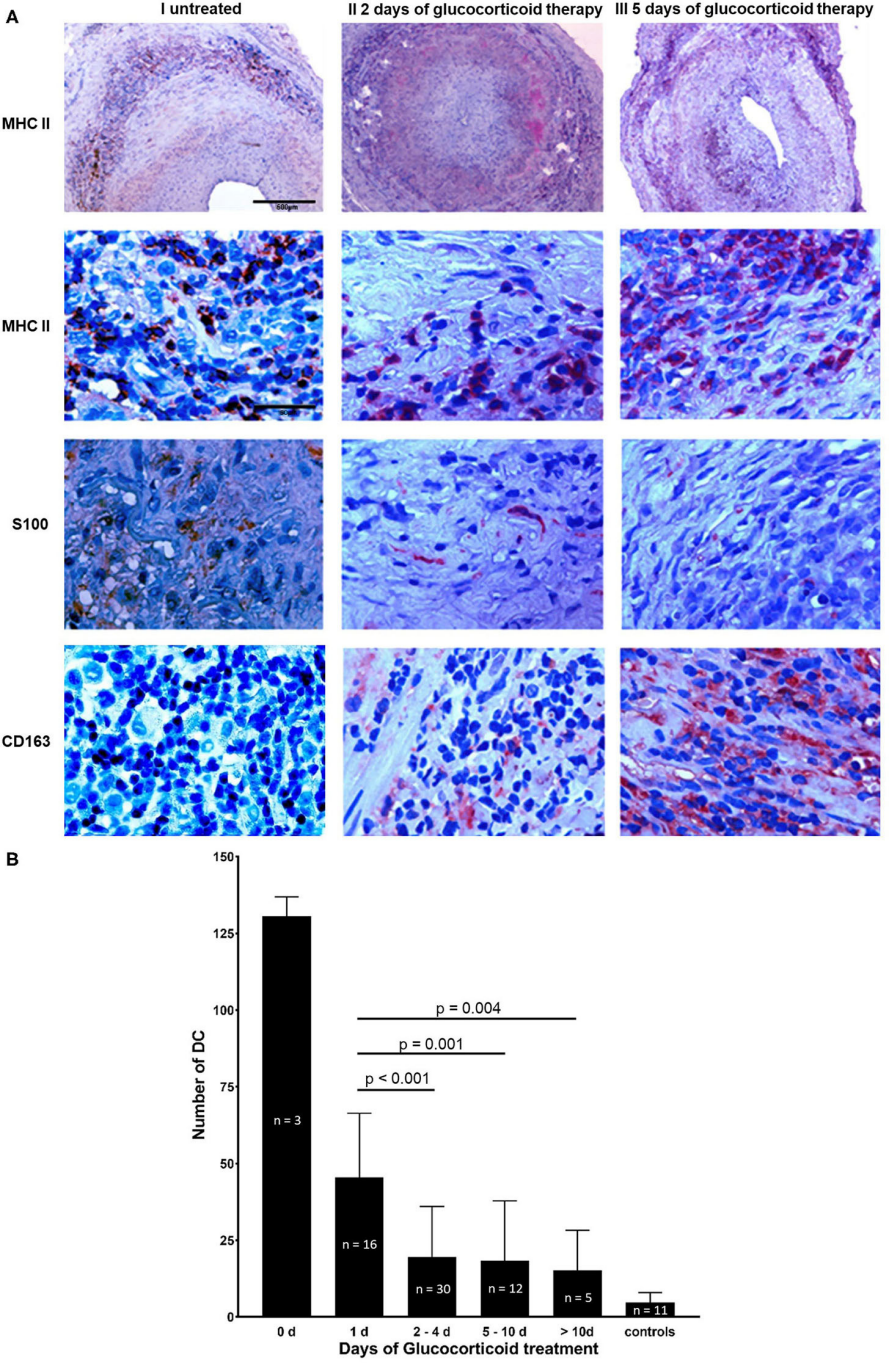
Reduction of DC under glucocorticoid therapy. **(A)** Temporal artery biopsy sections from three individual patients with GCA. Cross sections from a newly diagnosed untreated GCA patient are shown in column I, sections of a GCA patient after 2 days of glucocorticoid therapy are depicted in column II and tissue sections of a GCA patient 5 days after the initiation of glucocorticoid treatment are demonstrated in column III. Cross sections were stained with anti-MHC II mAb (row 1, 2), anti-S100 Ab (row 3), and anti-CD163 mAb (row 4). Cells positive for the particular epitope are indicated by red stain. Nuclei were stained with haematoxylin and eosin. Scale bar = 500 μm in row 1, scale bar = 50 μm in row 2–4. **(B)** Number of S100+ DC in 75 paraffin-embedded consecutive temporal artery sections from 41 GCA patients and 11 control patients. For each patient and control patient, S100+ cells were counted in three different temporal artery sections at 40 × magnification. Patients were grouped depending on the duration of treatment (0, 1, 2–4, 5–10, >10, controls).

The majority of DCs identified by immunohistochemical studies using anti-S100 Ab were located in granulomatous infiltrates (MHC II staining), in the adventitial layer and in a circular array along the internal and external elastic lamina (Figure 1A). In different temporal artery specimens DCs appeared in clusters. An inverse relation was observed between the length of glucocorticoid therapy and DC number (Figure 1B). Temporal arteries from both sides of an untreated GCA patient were examined. 135 DCs were present in a cross section of the right temporal artery, and 128 DCs in the cross section of the left side. After 1 day of glucocorticoid therapy 46 ± 21 DCs (mean ± standard deviation, *n* = 16 temporal arteries) were present in one tissue cross section. After 2–4 days only 20 ± 16 DCs (*n* = 30, *p* < 0.001) were detectable. Further decrease was monitored at later timepoints. Under long-term glucocorticoid therapy (>10 days) 9 ± 3 DCs (*n* = 5 temporal arteries) were determined (*p* = 0.004) in comparison to patients after 1 day of glucocorticoid treatment. An increase of monocyte-macrophage scavenger receptor CD163 was observed under glucocorticoid therapy that inversely correlates with the number of S100+ cells and the duration of glucocorticoid exposure (Figure 1A).

### Glucocorticoid Therapy Induces Apoptosis

**Figure 2:**
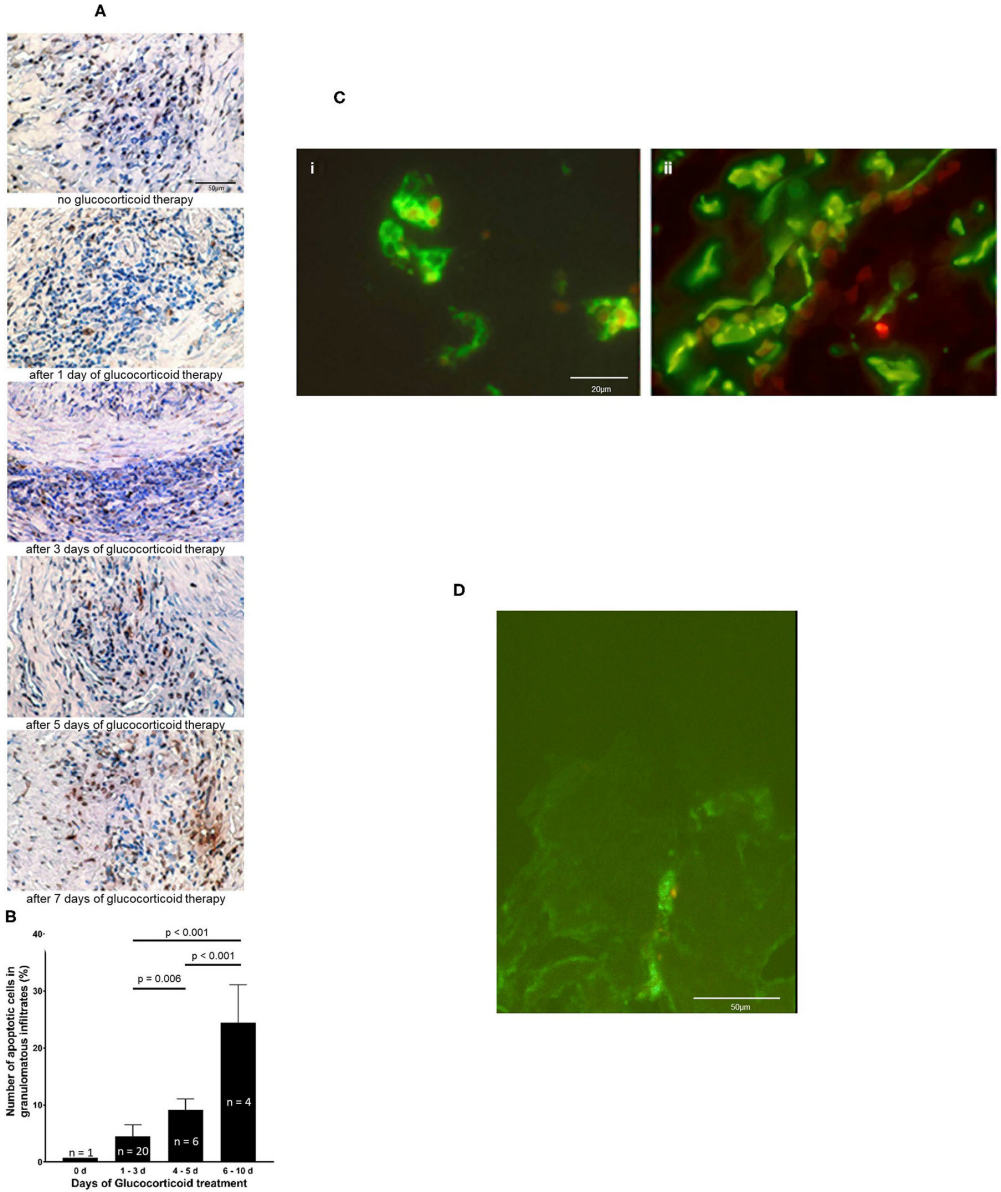
Glucocorticoid therapy induces apoptosis. **(A)** DNA fragmentation of apoptotic cells (brown stain) is demonstrated by applying TUNEL assay on temporal artery sections of five GCA patients. Nuclei were stained with haematoxylin and eosin. Scale bar = 50 μm. **(B)** The number of TUNEL-positive cells was quantified in 31 paraffin-embedded consecutive temporal artery specimens. For each patient and control patient, TUNEL-positive cells and nuclei of all cells in four random fields (magnification 100 × ) of each specimen were counted. The percentages of TUNEL-positive cells of all cells in the samples were calculated. **(C)** Demonstration of intracellular DNA fragmentation in CD3+ cells (i) and CD68+ cells (ii) in a temporal artery biopsy specimen from a patient with GCA 5 days after glucocorticoid treatment. T cells and macrophages are stained with anti-CD3 mAb and anti-CD68 mAb (green fluorescene), respectively. Applying the ApopTag Red Kit, rhodamine-labeled DNA fragmentation (red fluorescene) was identified. Scale bar = 20 μm. **(D)** Demonstration of intracellular DNA fragmentation in tissue-residing DC in a temporal artery biopsy specimen from a patient with GCA 5 days after glucocorticoid treatment. Confocal microscopy reveals rhodamine-labeled DNA fragmentation (red fluorescence) within DC, that is stained with anti-S100 Ab (green fluorescence). Scale bar = 50 μm.

An increase of apoptotic cells determined by TUNEL assay was seen with prolonged glucocorticoid therapy (Figure 2A). Glucocorticoid therapy induces apoptosis predominantly in inflammatory cell infiltrates. The percentage of apoptotic cells rose after 4–5 days of glucocorticoid to 9 ± 2%. From day 6 to day 10 glucocorticoids led to a significant increase in the amount of apoptotic cells, 27 ± 5% (*p* < 0.001, compared to the earlier timepoints) (Figure 2B). Double immunofluorescence demonstrated that CD3+ T cells and CD68+ macrophages (Figure 2C) underwent apoptosis. Confocal microscopy confirmed that DNA-fragments are intracellularly located in apoptotic S100+ DC. One example of intracellular DNA-fragmentation in DC is depicted in Figure 2D and in Supplementary Material Presentation.

### Reduction of CCL19 and CCL21 Expression in the Temporal Artery Specimens

**Figure 3:**
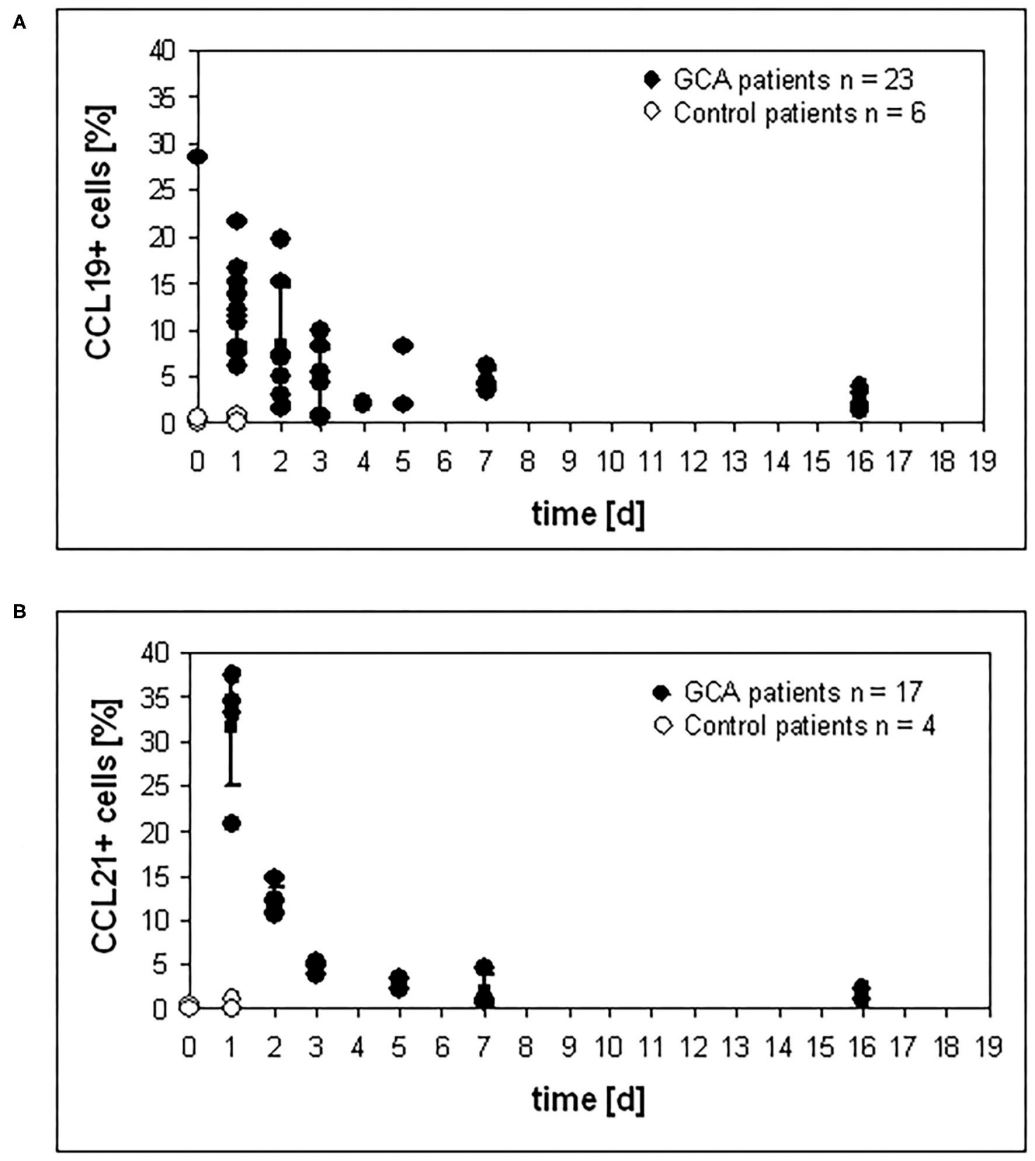
Reduction of CCL19 and CCL21 expression under glucocorticoid therapy. Temporal artery sections of patients were stained with **(A)** anti-CCL19 mAb and **(B)** anti-CCL21 mAB, respectively, and numbers of positive cells were determined in three sections. For each patient and control patient, positive cells were counted in three different temporal artery sections.

CCL19 and CCL21 serve as ligands for CCR7, which is prominently expressed on mature DC and the CCL19/21 – CCR7 system balances immunity and tolerance (28). To investigate the surface expression of both molecules on cells of the granulomatous infiltrates, immunohistochemistry was performed on temporal artery specimens of glucocorticoid-untreated and treated GCA patients and patients negative for GCA (controls). Compared to untreated control patients, high levels of CCL19+ cells in granulomatous infiltrates were observed in untreated GCA patients. After the onset of glucocorticoid therapy, the percentage of CCL19+ cells started to decrease. Continuous reduction was observed with increased duration of therapy (Figure 3A). The same tendency was observed for the percentage of CCL21+ cells (Figure 3B).

### Granulocyte-Macrophage Colony Stimulating Factor (GM-CSF) Expression Decreases Under Glucocorticoid Therapy

**Figure 4:**
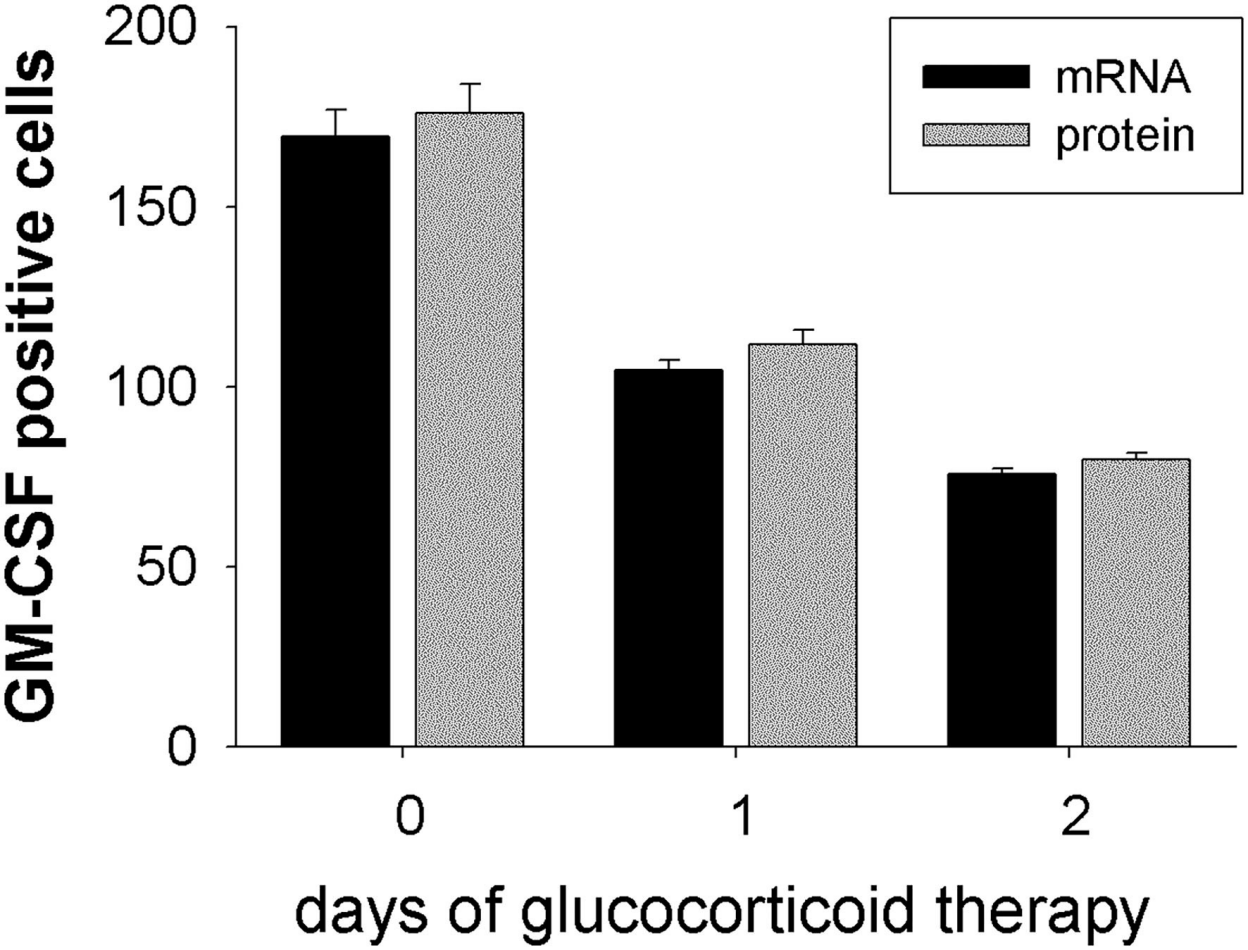
GM-CSF expression is down-regulated under glucocorticoid therapy. GM-CSF expression was determined on mRNA level by *in situ* hybridization (black bars) and on protein level by immunohistochemistry using anti-GM-CSF mAb (gray bars). Temporal artery biopsy specimens were obtained from GCA patients without glucocorticoid treatment (0 days; *n* = 3), or after 1 day of therapy (*n* = 10), or after 2 days of therapy (*n* = 9). For each patient GM-CSF+ cells were counted in two different temporal artery sections at 40 × magnification.

Expression of GM-CSF in temporal artery biopsy specimens from GCA patients that where either not treated (0 days), or 1 day or 2 days treated, was analyzed by *in situ* hybridization on mRNA level and by immunohistochemistry on protein level. Both techniques delivered concurrent results for the expression of GM-CSF+ cells. In untreated patients about 172 GM-CSF+ cells were detected (mRNA: 169 ± 7.4, protein: 176 ± 7.9; *n* = 3) in the arterial cell wall. Already after 1 day of glucocorticoid treatment a reduction of GM-CSF+ cells was observed (mRNA: 104 ± 2.9, protein: 112 ± 4.0; *n* = 10). After 2 days of treatment <50% GM-CSF+ cells (mRNA: 76 ± 1.6, protein: 80 ± 1.9; *n* = 9) were detected, as compared to untreated patients (Figure 4). A significantly reduced CSF2RB expression was observed in peripheral blood CD1c+ DCs (Table 1).

**Table 1:** Glucocorticoid therapy reduces CSF2RB expression in peripheral blood CD1c+ DCs.

	**Mean Normalized Expression ( × 10.000)**	
	**Day 0**	**Day 2**
CSF2RB	1914.12	0.50

## Discussion

Administration of glucocorticoids suppresses the accumulation of DCs and leads to a rapid, time-dependent normalization of DCs in temporal arteries from GCA patients. The findings reported here suggest that this effect is mediated by induction of apoptosis since the percentage of apoptotic cells increased continuously according to the length of glucocorticoid application. TUNEL positive cells were mainly located in the inflammatory lesions. Apoptosis could be the central mechanism reducing DC and other inflammatory cells in temporal arteries in GCA. Interestingly, mature DCs express a pro-apoptotic glucocorticoid receptor isoform, which is not present in immature DCs (29). Therefore, we conclude that glucocorticoids effectively suppress inflammation in GCA by diminishing cell-mediated immunity. Exposure of macrophages to glucocorticoids for 24 h specifically enhances the uptake of apoptotic leukocytes by both human and murine macrophage population (30). CD163 is used as *in vivo* marker for alternatively activated macrophage (31). Its expression is suppressed by pro-inflammatory mediators such as LPS, IFN-γ and TNF-α, whereas IL-6 and the anti-inflammatory cytokine IL-10 strongly up-regulate CD163 expression (32). Moreover, CD163 mediates IL-10 release itself (33). The observed increase of CD163 in temporal artery biopsy specimens from glucocorticoid treated GCA patients further supports our conclusion.

In many cell types, glucocorticoid receptor activation leads to G_1_ cell cycle arrest, this cytostatic condition is often reversible, such that upon withdrawal of glucocorticoid the cells re-enter the cell cycle (34). In other cell types, glucocorticoid treatment is cytotoxic and irreversible and results in programmed cell death or apoptosis (35). The transcriptional regulatory mechanisms underlying the cytostatic vs. the cytotoxic effects of the glucocorticoid receptor and the target genes affected by the receptor need to be determined. In summary, the described glucocorticoid-induced apoptosis of DC, macrophages and T cells in the granulomatous lesions seem to be an important mechanism for the immunosuppressive effect and especially the control of autoreactivity and autoimmunity.

In contrast, disease relevant tissue residing IL-12-IFN-γ-producing Th1 cells are most likely being protected from apoptosis. Plasma levels of IFN-γ are elevated in untreated GCA cases and remain elevated after corticosteroid therapy (1). Accordingly, these cells can cause disease relapse after discontinuation of GC treatment (6). This might be explained by the fact that in some cell types glucocorticoid receptor activation leads to G1 cell cycle arrest. This cytostatic condition is reversible, such that upon withdrawal of glucocorticoid therapy the cell re-enters the cell cycle (34). In other cell types, glucocorticoid treatment is cytotoxic and irreversible resulting in programmed cell death or apoptosis (35).

Another important mechanism detected in our study is the inhibition of leukocyte migration. CCL19 and CCL21 lead to migration of mature DC into lymphatic tissues for antigen presentation (26). It was hypothesized that granulomatous infiltrates in temporal arteries of GCA patients serve as a “pathologic” lymphatic tissue (36). Reduced expression of CCL19 and CCL21 under glucocorticoid therapy could thus explain reduced numbers of DCs in the granulomatous lesions by inhibition of migration and activation of DCs. Glucocorticoids also suppress the trafficking of immune cells *in vivo* by increasing the protein expression of macrophage migratory inhibitory factor (37).

Under inflammatory conditions, activated CD4+ T-helper cells produce large amounts of GM-CSF at systemic level. In this way an emergency myelopoiesis drives macrophages and dendritic cells or common monocyte precursors into cell cycle and releases increased amounts of classical monocytes into the blood. In peripheral blood these monocytes again differentiate into monocyte-derived dendritic cells. It is known that GM-CSF controls some common and peripheral dendritic cell functions (38).

In tissue samples from GCA patients GM-CSF was predominantly expressed in the adventitial layer, where we also detected the granulomatous infiltrates. Under glucocorticoid therapy we found a significant reduction of GM-CSF+ cells in the arterial wall. As this factor induces proliferation and activation of various types of leukocytes, the downregulation of GM-CSF might also contribute to the observed decrease of DC numbers in GCA under glucocorticoid treatment. Secondly, GM-CSF is a locally expressed mediator that has the capacity of recruiting circulating leukocytes. Real-time PCR of peripheral CD1c+ DCs in GCA patients revealed a down-regulation of CSF2RB. CSF2RB is known as the common beta-subunit of GM-CSF, IL-3 and IL-5-receptors (39). Glucocorticoid therapy may therefore prevent the injured artery in GCA from recruiting further DCs. This conclusion is also in line with decreasing numbers of DCs in the examined biopsy specimens. Furthermore, GM-CSF has additional angiogenic properties (40). Blockade of GM-CSF by GC therapy might inhibit angiogenesis of the vasa vasorum in GCA and thereby reduce leukocyte trafficking.

Granulocyte colony-stimulating factor (G-CSF) and granulocyte-macrophage colony-stimulating factor (GM-CSF) have proinflammatory activities. In mouse models of rheumatoid arthritis it was shown that antagonism of G-CSF or GM-CSF significantly reduced disease activity (41). In addition, it was shown that G-CSF and GM-CSF administration can exacerbate rheumatoid arthritis, and their antagonism has a potential to reduce disease activity in RA (42). Therefore, we hypothesize, that antagonism of G-CSF or GM-CSF might also be an effective way of treating other inflammatory disorders, such as GCA. We strongly hope that our data support the initiation of a clinical trial regarding GM-CSF antagonization in GCA.

In summary, the described glucocorticoid-induced apoptosis of DC, macrophages and T cells in the granulomatous lesions seem to be an important mechanism for the immunosuppressive effect in GCA. In future studies it will have to be evaluated whether other mechanisms contribute to this effect. Further understanding of these mechanisms may allow tailoring therapies that facilitate the resolution of the inflammatory process in GCA.

## Data Availability Statement

The original contributions presented in the study are included in the article/Supplementary Material, further inquiries can be directed to the corresponding author/s.

## Ethics Statement

The studies involving human participants were reviewed and approved by Ethikkommission der Medizinischen Hochschule Hannover. The patients/participants provided their written informed consent to participate in this study.

## Author Contributions

ADW and UW substantially contributed equally to conceptualization, formal analysis, resources, supervision, and writing of the original draft. JT and JH substantially contributed to formal analysis, investigation, and reviewing and editing of the manuscript. TW contributed to the preparation of the tables and editing of the manuscript. VG and SR substantially contributed to project administration and reviewing and editing of the manuscript. MZ substantially contributed to conceptualization, project administration, supervision, and reviewing and editing of the manuscript. All the authors revised the paper and approved the final version of the article to be published.

## Funding

This work was partially funded by a grant from the Deutsche Forschungsgemeinschaft (DFG, German Research Foundation) WA 747/4-1 (ADW).

## Conflict of Interest

The authors declare that the research was conducted in the absence of any commercial or financial relationships that could be construed as a potential conflict of interest.

## Publisher's Note

All claims expressed in this article are solely those of the authors and do not necessarily represent those of their affiliated organizations, or those of the publisher, the editors and the reviewers. Any product that may be evaluated in this article, or claim that may be made by its manufacturer, is not guaranteed or endorsed by the publisher.
