# Supplementary material for: Glucocorticoid Effects on Tissue Residing Immune Cells in Giant Cell Arteritis: Importance of GM-CSF
Source: Front Med (Lausanne). 2021 Sep 7;8:709404. doi: 10.3389/fmed.2021.709404 (PMC8452956; doi:10.3389/fmed.2021.709404)
Supplement: Supplementary file 2 [file Table_2.DOCX]

| Primer name | Primer 1 | Primer 2 | Design/  Manufacturer |
| --- | --- | --- | --- |
| CSF2RB | n.k. | n.k. | Superarray  PPH01137A |
| RPS9 | GGCGCAGACGGGGAA | GGGTCACATAAGTTTTGCGACAA | (31)/MWG Biotech |

**Supplementary Table 2**. Primer used for real-time PCR on RNA isolated from CD1c+ DC of GCA patients.
