# Supplementary figures and images for: Glucocorticoid Effects on Tissue Residing Immune Cells in Giant Cell Arteritis: Importance of GM-CSF
Source: Front Med (Lausanne). 2021 Sep 7;8:709404. doi: 10.3389/fmed.2021.709404 (PMC8452956; doi:10.3389/fmed.2021.709404)

## Slide 1
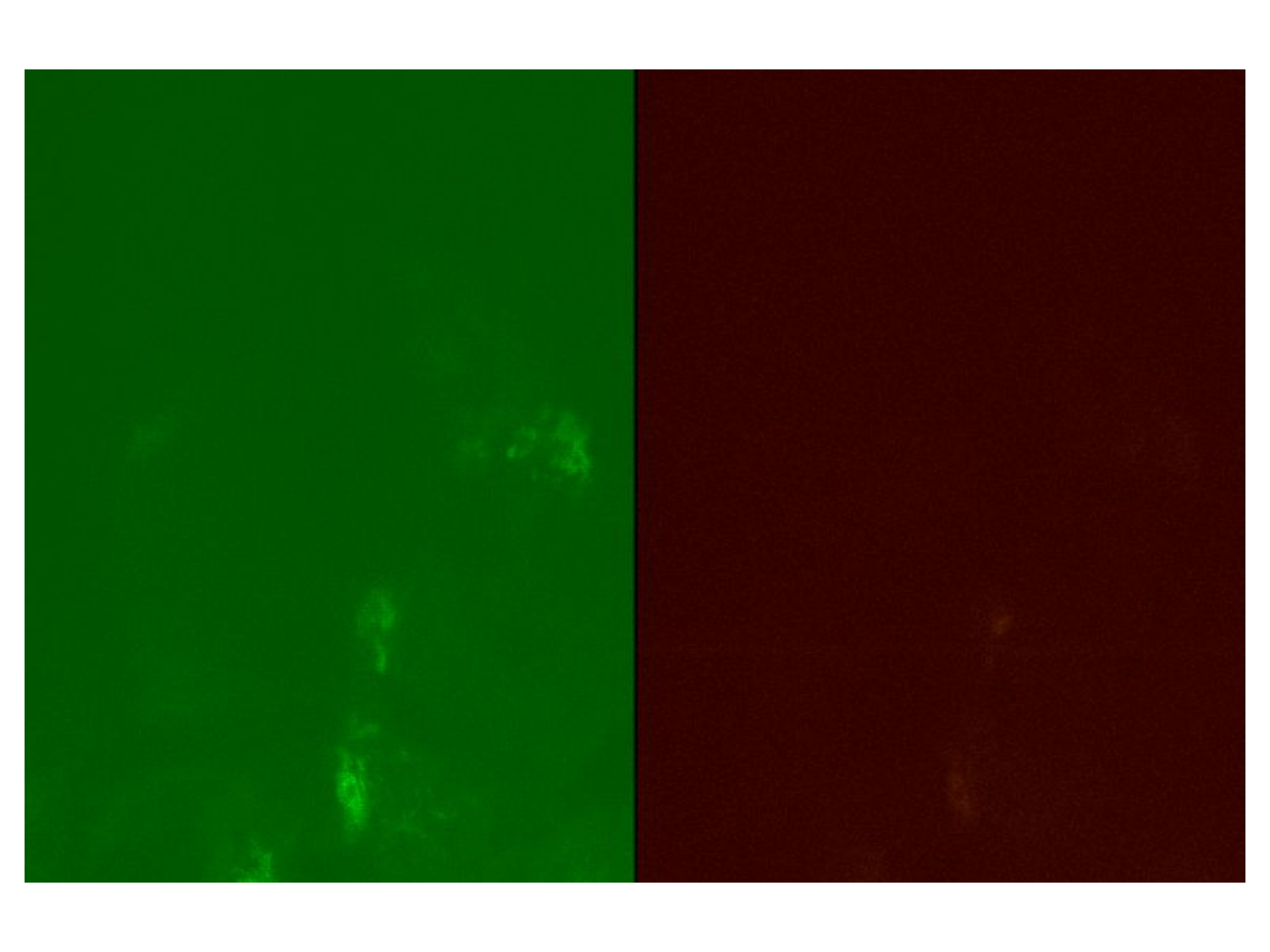

Supplement: Supplementary file 3 [file Presentation_1.PPT]
